# Interatrial Shunt Devices in Heart Failure: A Therapy in Search of the Right Patient

**DOI:** 10.3390/jcdd13100519

**Published:** 2026-10-16

**Authors:** Asimina Karagianni, Alexandros Evangeliou, Dimitrios Orestis Pavlou, Vaios Schismenos, Efstathios Pagourelias, Georgios Giannopoulos, Christodoulos Papadopoulos, Theodoros Karamitsos, Vassilios Vassilikos, Stergios Tzikas

**Affiliations:** 3rd Department of Cardiology, Aristotle University of Thessaloniki, 54626 Thessaloniki, Greece

**Keywords:** interatrial shunt, heart failure with preserved ejection fraction, heart failure with mildly reduced ejection fraction, left atrial decompression, pulmonary capillary wedge pressure, responder phenotype

## Abstract

Heart failure with preserved or mildly reduced ejection fraction (HFpEF/HFmrEF) accounts for approximately half of the global heart failure burden and remains a syndrome with limited device-based therapeutic options. It is often characterized by a disproportionate rise in left-sided filling pressure during exercise. Interatrial shunt devices (IASDs) aim to reduce left atrial pressure through controlled left-to-right atrial communication. We performed a structured narrative review of mechanistic studies, early feasibility cohorts, randomized trials, meta-analyses, and ongoing studies indexed through to August 2026. Early proof-of-concept studies consistently demonstrated technical feasibility, durable patency, and favorable hemodynamic effects, particularly through reductions in pulmonary capillary wedge pressure (PCWP), often accompanied by improved functional status. However, larger sham-controlled randomized trials failed to show consistent overall benefit in unselected populations. Recent mechanistic and observational data indicate that pulmonary vascular reserve, right ventricular–pulmonary arterial coupling, rhythm-device status, and device design may affect benefits and harms. Latent pulmonary vascular disease and impaired right-heart reserve may identify patients poorly suited for shunt implantation, whereas carefully selected, promising phenotypes remain under prospective study. IASDs should therefore remain investigational. Future trials should use exercise hemodynamics and validated noninvasive markers to prospectively define responders, assess device-specific effects, and reconcile physiological unloading with clinical outcomes.

## 1. Introduction

Heart failure with preserved ejection fraction (HFpEF) remains a leading cause of mortality and morbidity and—along with heart failure with mildly reduced ejection fraction (HFmrEF)—makes up about 50% of heart failure cases [1]. Considering the aging population and accumulation of cardiometabolic risk factors, such as obesity, diabetes, and hypertension, the prevalence of HFpEF is increasing.

Unlike heart failure with reduced ejection fraction (HFrEF), for which several disease-modifying treatments have been established, treatment options for HFpEF remain comparatively limited. Guideline-based management emphasizes use of diuretics for congestion, treatment of comorbidities, lifestyle optimization, and, in selected patients, mineralocorticoid receptor antagonists (MRAs), angiotensin receptor–neprilysin inhibitors (ARNIs), and sodium–glucose cotransporter-2 (SGLT2) inhibitors [2]. Suboptimal outcomes and the marked pathophysiologic heterogeneity of HFpEF have prompted growing interest in phenotype-directed interventions.

One such intervention is the implantation of an interatrial shunt device (IASD). The rationale for this strategy is based on a central hemodynamic abnormality seen in many HFpEF and HFmrEF phenotypes: a disproportionate rise in left-sided filling pressures during exercise, secondarily transmitted to the pulmonary circulation. Controlled left-to-right atrial communication aims to decompress the left atrium, reduce pulmonary venous congestion, and improve symptoms.

The trajectory of interatrial shunt therapy mirrors that of several previous HFpEF interventions, in which promising mechanistic and hemodynamic signals did not translate into clinical benefit when applied to broad patient populations. This experience reinforces the view that HFpEF is not a single disease entity but rather a syndrome composed of multiple pathophysiological phenotypes requiring individualized therapeutic approaches. Emerging evidence suggests that the success of interatrial shunting depends less on the device itself and more on identifying the appropriate hemodynamic patient phenotype.

This narrative review critically synthesizes the pathophysiological rationale, device-specific evidence, randomized trials, and emerging responder and risk phenotypes for interatrial shunt therapy across the ejection-fraction spectrum.

## 2. Pathophysiological Rationale

HFpEF is a heterogeneous syndrome involving multiple interacting cardiac, vascular and extracardiac pathophysiological mechanisms, including impaired left ventricular relaxation, increased left ventricular stiffness, myocardial fibrosis, microvascular inflammation, endothelial dysfunction, abnormal ventricular–vascular coupling and chronotropic incompetence [1].

A core identified pathophysiological abnormality is the characteristic disproportionate rise in left ventricular (LV) filling pressure during exercise, due to impaired ventricular relaxation and increased chamber stiffness. The resulting increase in left atrial pressure is retrogradely transmitted to the pulmonary capillary bed, producing exertional pulmonary congestion and dyspnea, via a marked increase in pulmonary capillary wedge pressure (PCWP) during exercise. This exertional PCWP correlates with HFpEF’s symptom severity and more specifically clinical manifestations of dyspnea and decreased functional capacity, even when resting LV filling pressures are normal or only mildly elevated.

Clinical observations have long supported the concept that left atrial decompression may mitigate symptoms. In Lutembacher syndrome, the coexistence of mitral stenosis and an atrial septal defect (ASD) may attenuate left atrial pressure and pulmonary congestion. Moreover, while the closure of congenital ASDs is considered safe practice, ASD closure may precipitate pulmonary edema in patients with subclinical LV dysfunction, which can be reversed with ASD re-establishment or fenestration.

These observations led to the hypothesis [3] that the creation of a small-diameter atrial shunt in HFpEF patients with elevated left ventricular filling pressures—and thus increased exertional PCWP—would alleviate symptoms developed due to pulmonary congestion. The shunt is thought to allow for partial left atrial (LA) pressure decompression into the right atrium, potentially decreasing PCWP and pulmonary venous pressures at rest and particularly during activity. This physiological unloading of the left atrium forms the conceptual basis for therapeutic interatrial shunting and the development of IASDs.

Interatrial shunting inevitably increases right atrial pressure, right ventricular preload, and pulmonary blood flow. The therapeutic premise of IASDs therefore relies on a dynamic balance between effective left atrial decompression and the capacity of the right ventricle and pulmonary circulation to accommodate the additional volume load.

Early hemodynamic modeling and computational simulations suggest that a shunt diameter of approximately 8 mm, producing a pulmonary-to-systemic flow ratio (Qp/Qs) of about 1.3 to 1.4, could provide meaningful left atrial decompression with minimal right-sided volume overload [4]. However, this balance is likely to vary substantially across devices and patients, and is especially dependent on patients’ pulmonary vascular reserve and right ventricular function. For context, congenital atrial communications are commonly considered hemodynamically significant when Qp/Qs is at least 1.5 [5].

Subsequent clinical trials demonstrated that the pulmonary vascular response during exercise is a critical determinant of tolerance to shunt therapy. When pulmonary vascular resistance (PVR) remains low or decreases appropriately, the pulmonary circulation and right ventricle may accommodate the IASD-mediated additional flow. By contrast, when PVR is abnormally elevated, shunting simultaneously increases right ventricular preload and afterload, potentially worsening tricuspid regurgitation, right ventricular dilation, and venous congestion and increasing the risk of heart failure and adverse clinical outcomes.

**Figure 1:**
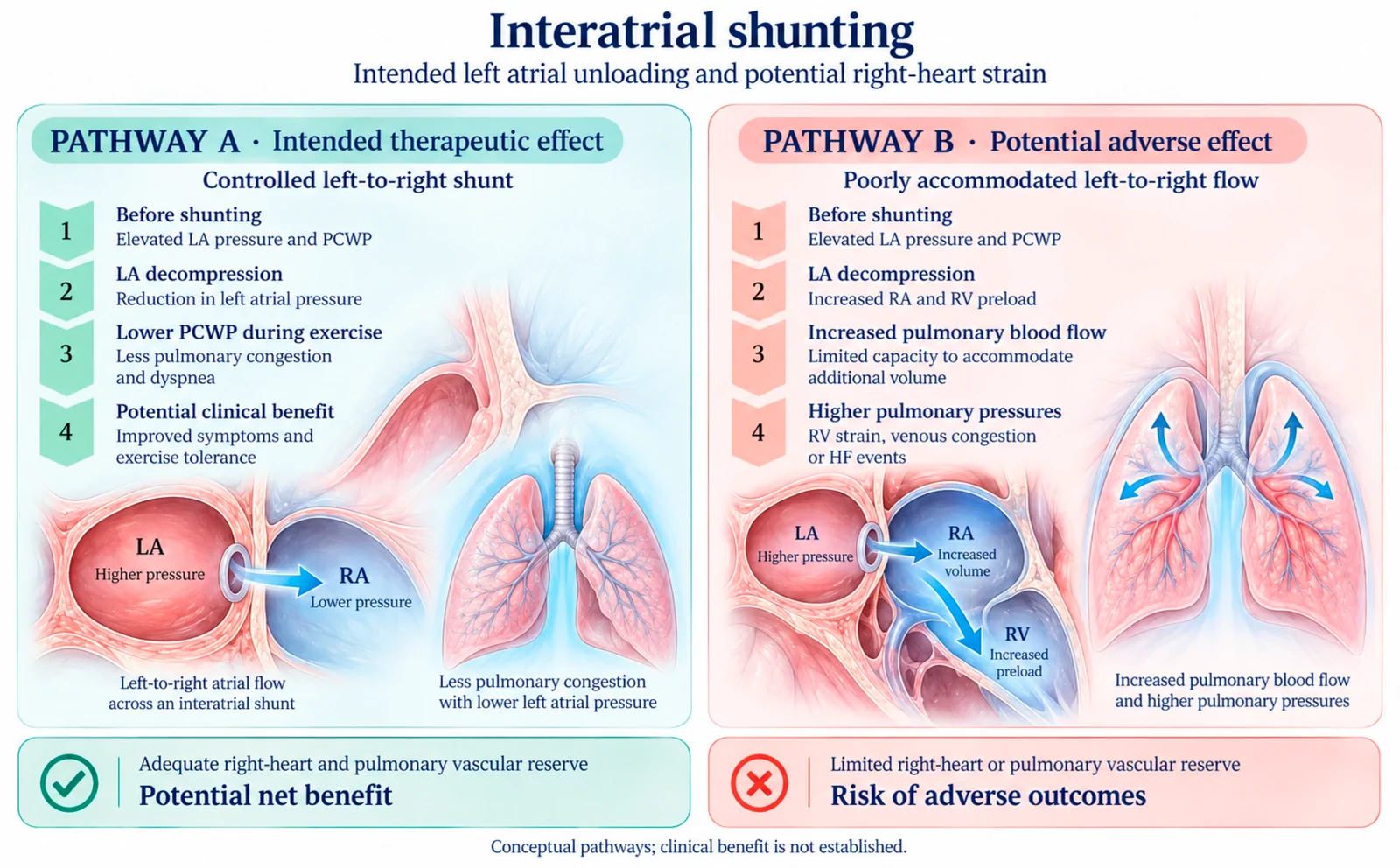
Hemodynamic consequences of interatrial shunting. Controlled left-to-right shunting from the left atrium (LA) to the right atrium (RA) can reduce LA pressure and pulmonary capillary wedge pressure (PCWP) (PATHWAY A). Conversely, an increase in pulmonary blood flow and right ventricular (RV) preload may lead to adverse clinical outcomes when pulmonary vascular or right-heart reserve is limited (PATHWAY B).

These considerations are particularly relevant in patients with latent pulmonary vascular disease (PVD), a phenotype characterized by relatively preserved resting pulmonary hemodynamics but an abnormal increase in exercise PVR. Such patients may be poorly suited to shunting despite apparently favorable resting measurements. This concept has become central to current attempts to refine patient selection for IASD implantation [6]. Figure 1 summarizes the intended therapeutic effects and the competing risk of right-heart strain.

Although IASDs were initially developed to aim specifically at HFpEF’s pathophysiology, multiple early feasibility studies and one major randomized trial (RELIEVE-HF) have included both HFmrEF/HFpEF and HFrEF patients. Device design, shunt size, and implantation eligibility criteria vary considerably across devices, making class-wide assumptions inappropriate.

## 3. Search Strategy

We conducted a structured narrative search of PubMed/MEDLINE, the Cochrane Library and ClinicalTrials.gov from database inception through August 2026. Search terms combined “interatrial shunt”, “atrial shunt device”, “IASD”, “left atrial decompression”, “heart failure”, “HFpEF”, “HFmrEF”, and “HFrEF” with device and trial names where appropriate. Randomized trials, prospective cohorts, registries, early feasibility studies, relevant mechanistic investigations, and meta-analyses in human heart failure populations were included.

Titles and abstracts were screened for relevance, followed by full-text review of eligible articles. Editorials and commentary articles were reviewed to contextualize primary evidence. Reference lists of key publications were screened to identify additional relevant studies. Narrative reviews, case reports, and studies focusing on non-heart failure indications were excluded.

Because this article is a narrative rather than a systematic review, no protocol was registered, no formal risk-of-bias instrument was applied, and no de novo meta-analysis was performed. Findings are therefore synthesized qualitatively, with emphasis on study design, device-specific limitations, and the distinction between exploratory and confirmatory evidence.

## 4. Clinical Evidence for Interatrial Shunt Devices

Atrial shunt therapy has evolved from proof-of-concept septal implants to a broader field of left atrial decompression systems. Evidence is most mature for the Corvia IASD and V-Wave systems, with additional early experience involving the Occlutech Atrial Flow Regulator (AFR), no-implant transseptal systems, and left atrial-to-coronary sinus shunts. The principal IASDs under clinical investigation differ considerably in engineering design, fenestration diameter, intended clinical application, and current stage of clinical development, as summarized in Table 1. Additionally, the structural designs of the IASDs discussed in the review are illustrated in Supplementary Figure S1.

### 4.1. Trial Eligibility, Natriuretic Peptides and Post-Procedural Antithrombotic Therapy

In the REDUCE LAP-HF randomized trials (I and II), IASD implantation was investigated in patients with symptomatic HFpEF or HFmrEF despite optimal guideline-directed medical therapy and an NYHA functional class III or ambulatory IV [7,8,9]. Eligibility criteria included objective evidence of elevated LA pressure during exercise (≥25 mmHg) and a PCWP-RA pressure gradient ≥5 mmHg. Other inclusion requirements were either a prior HF hospitalization or elevated B-type natriuretic peptide (BNP) or N-terminal pro-BNP (NT-proBNP). Several factors that could increase procedural or hemodynamic risk, including stage D HF, low (<2.0 L/min/m^2^) cardiac index, significant RV dysfunction, elevated resting RAP, and markedly increased PVR, have been used as exclusion criteria. These criteria were protocol-specific and should not be interpreted as universally applicable indications for IASD implantation. Although initial IASD studies primarily focused on HFpEF and HFmrEF, patients with HFrEF have also been included in mixed-EF investigations, including AFR-PRELIEVE and RELIEVE-HF. In the RELIEVE-HF randomized trial, inclusion criteria included an NYHA class II, III or ambulatory IV, despite guideline-directed therapy [10]. It should be highlighted that no evidence-based routine indication for shunt implantation in HFpEF/HFmrEF or HFrEF has been established by the reviewed randomized trials. Clinical trial eligibility should therefore be clearly separated from an approved device indication or guideline recommendation.

One key patient inclusion criterion in the trials was the elevation of baseline BNP or NT-proBNP, which is a relevant biomarker for evaluation of myocardial wall stress post shunt implantation. However, it was not consistently reported as a comparative efficacy outcome across the major randomized IASD trials. Consequently, the available evidence does not permit a reliable assessment of whether IASD implantation produces a reproducible treatment-related reduction in natriuretic peptide concentrations or whether changes in these biomarkers are associated with clinical benefit.

Post-procedural antithrombotic treatment varied across studies and devices. Permanent implant-based shunts are generally managed with dual antiplatelet therapy for 3–6 months, followed by long-term aspirin, whereas patients with an established indication for oral anticoagulation are managed accordingly. Implant-free systems required different regimens. The optimal antithrombotic regimen has not been verified and remains undetermined.

### 4.2. Proof-of-Concept and Early Feasibility Studies

#### 4.2.1. Corvia IASD: First-in-Human Feasibility

The first formal proof-of-concept evaluation of a purpose-built interatrial shunt in HFpEF/HFmrEF was the 2012 Corvia pilot study [11]. In this non-randomized study, implantation was successful in all 11 patients, with no major periprocedural device-related complications. Early follow-up suggested lower PCWP without major adverse changes in right atrial pressure or pulmonary artery systolic pressure, along with improvements in exercise capacity and quality-of-life metrics. The 1-year follow-up confirmed shunt patency and sustained improvements in symptoms, health status, and resting PCWP, with no late device-related complications [12].

#### 4.2.2. Occlutech AFR: Pilot and Early Clinical Experience

The Occlutech Atrial Flow Regulator (AFR) has demonstrated the widest range of clinical applications, which can be partly attributed to the flexibility of the device’s design and its multiple fenestration diameters, allowing for different degrees of atrial decompression. Beyond its investigational use in heart failure, it has been utilized in severe pulmonary arterial hypertension (PAH) [13], in pediatric populations with congenital heart disease [14] and in patients with restrictive cardiomyopathy [15]. When intended for PAH, the created shunt is smaller to allow for controlled right-to-left atrial decompression with minimal systemic desaturation, whereas large fenestrations (typically around 8 mm) are used in HFpEF patients to facilitate left-to-right flow and left atrial unloading. This design flexibility distinguishes the AFR from fixed-diameter devices of about 8 mm developed solely for heart failure.

AFR-PRELIEVE was an open-label, non-randomized, prospective pilot study of the AFR in symptomatic [16] NYHA class III–IV heart failure patients with either HFpEF/HFmrEF or HFrEF [17]. There was technical success of implantation in all 36 patients, with low rates of serious adverse events and confirmed shunt patency at 3 months. Pulmonary artery pressure did not increase significantly, and functional and clinical measures improved.

The extended AFR-PRELIEVE cohort included 53 patients [18]. At 3 months, resting PCWP decreased, with a sustained signal in the HFpEF subgroup analysis. At 12 months, right ventricular function remained stable, and pulmonary artery pressure did not increase. Clinical evaluation suggested sustained overall improvements in NYHA functional class and quality-of-life measures, and in both the HFpEF and HFrEF subgroups separately.

The PROLONGER study [19] and FLOW-HF program provide additional observational experience. ClinicalTrials.gov listed the AFTER registry (NCT04405583) as recruiting as of August 2026, with longer-term data still pending.

A 2026 exploratory analysis of 106 participants of the PRELIEVE study found that lower baseline tricuspid annular plane systolic excursion/pulmonary artery systolic pressure (TAPSE/PASP), a marker of impaired right ventricular–pulmonary arterial coupling, was associated with a greater recurrent heart failure hospitalization burden; this post hoc association requires prospective validation [20].

#### 4.2.3. V-Wave: First- and Second-Generation Development

The first-in-human V-Wave study enrolled 10 patients with advanced HFrEF [21]. Technical success was achieved in all implanted patients, with no device-related adverse events and confirmed patency in all cases. Improvements in NYHA class and quality-of-life scores were observed, as well as significant reductions in resting PCWP, without significant changes in mean pulmonary artery pressure or right atrial pressure.

A subsequent multicenter study expanded eligibility to both HFrEF and HFpEF patients [22]. A total of 38 patients participated in the study and procedural success was achieved in 37. At 12 months, significant valve stenosis or occlusion occurred in a substantial proportion of patients, and only those with patent shunts demonstrated reductions in PCWP. Post hoc analysis demonstrated higher risk of death and heart failure hospitalization in patients with stenotic or occluded shunts, highlighting the importance of device durability and motivating subsequent design modification.

Given the high rates of valve stenosis and occlusion observed with the first-generation device, the principal modification implemented in the second-generation V-Wave was the removal of the device’s valve. In an initial 10-patient experience, implantation was technically successful in all, with one periprocedural death [23]. At 1 year, sustained shunt patency without evidence of narrowing was confirmed.

#### 4.2.4. Emerging and Implant-Free Technologies

The APTURE System (Edwards Lifesciences) creates left atrial-to-coronary sinus communication rather than a conventional transseptal shunt and should be considered within the left atrial decompression field. Early feasibility results have been reported [24] and larger randomized trials are underway. Implant-free systems designed to create controlled interatrial communication with no permanent prosthesis, such as the Alleviant [25], NoYA, and InterShunt PAS-C platforms, are also being evaluated in ongoing early feasibility and randomized studies. Evidence for HFrEF was expanded with SUSTAIN-HF, a prospective multicenter single-arm study of 120 patients treated with the D-Shant system [26]. At 1 year, D-Shant implantation was associated with improvements in functional capacity, quality-of-life measures, and left ventricular function.

#### 4.2.5. Encouraging Physiological Signals of Early Trials

**Figure 2:**
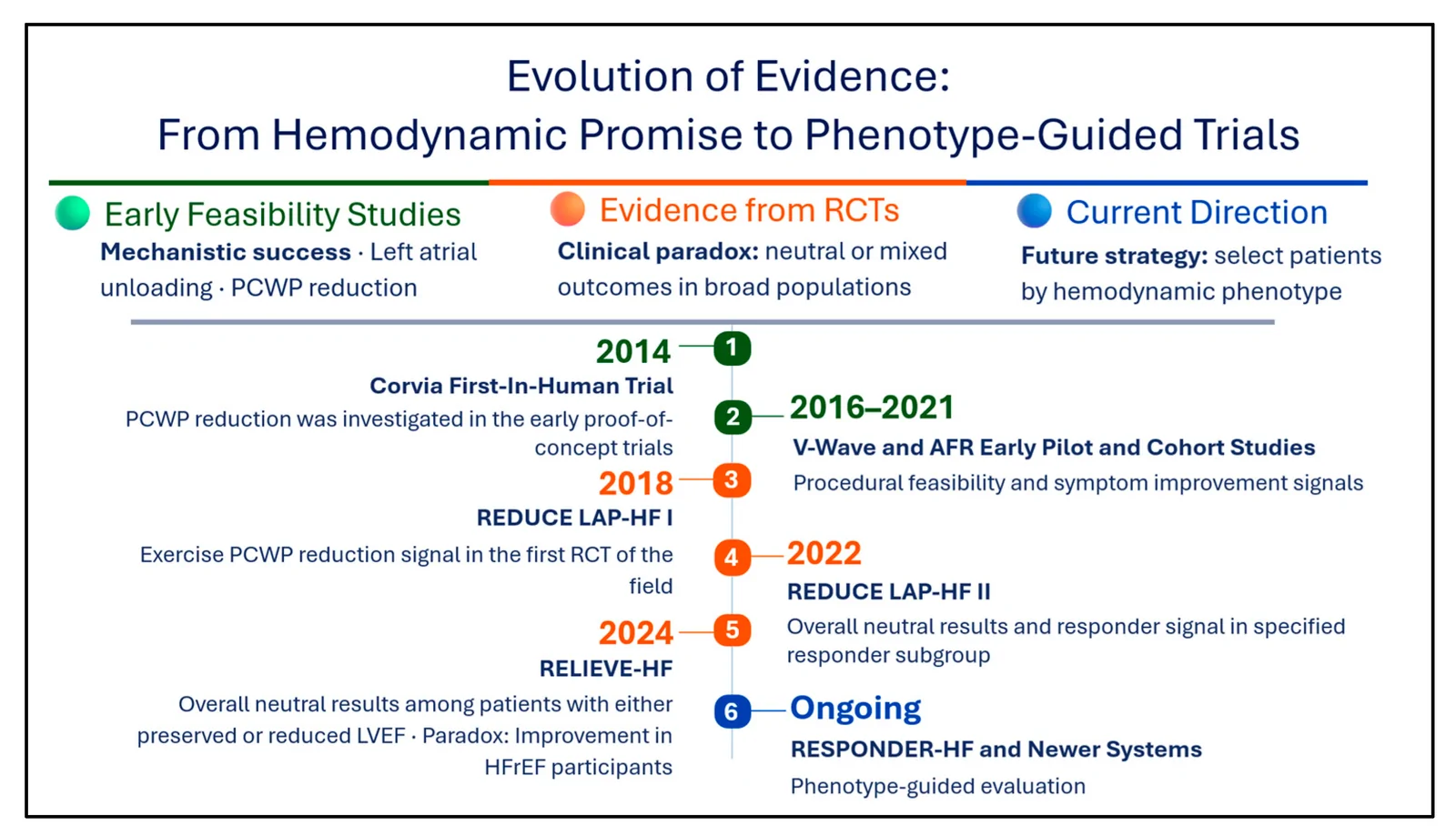
Chronological evolution of evidence for IASDs. Early studies consistently demonstrated feasibility and favorable physiological effects. Subsequent randomized controlled trials (RCTs) produced neutral overall results and shifted research focus toward phenotype-guided patient selection, responder analyses, and individualized hemodynamic characterization.

Collectively, early feasibility studies established the mechanistic validity of interatrial shunting by demonstrating procedural feasibility; reproducible reductions in PCWP, particularly during exercise; and parallel improvements in functional status and quality-of-life measures. Nonetheless, these findings derive predominantly from small, single-arm, non-randomized studies and are therefore susceptible to selection bias, placebo effects, and overestimation of benefit. This chronological evolution of IASD trials and clinical evidence is summarized in Figure 2.

### 4.3. Randomized Controlled Trials

#### 4.3.1. REDUCE LAP-HF I

REDUCE LAP-HF I was the first randomized, single-blind, sham-controlled evaluation of an IASD—specifically the Corvia IASD—in patients with HFpEF and HFmrEF [7]. The principal effectiveness measure was change in exercise PCWP 1 month after implantation. A primary safety endpoint was also evaluated, and secondary outcomes, including functional status and quality-of-life parameters, were measured at annual follow-ups.

Forty-four patients were randomized, 22 per group [27]. A statistically significant greater reduction in exercise PCWP was reported in the treatment group when compared with the control group, with no significant difference in the primary safety outcome between the groups.

At 1 year, shunt patency was confirmed in all patients with an IASD and no statistically significant difference in safety outcomes was demonstrated between the two arms of the trial [8]. Right ventricular size increased at 6 months in the treatment arm, while no further significant increase occurred at 12 months. Right ventricular ejection fraction, TAPSE and pulmonary artery systolic pressure remained unchanged. Regarding the secondary outcome measures at 12 months, there were no significant changes. Changes in New York Heart Association (NYHA) functional class, 6-Minute Walk Distance (6MWD), and quality-of-life parameters [Kansas City Cardiomyopathy Questionnaire—Overall Summary Score (KCCQ-OSS) and EuroQol 5-Dimension (EQ-5D) scale] were evaluated at 12 months as secondary outcomes. Although improvements from baseline were observed in some measures, between-group differences were not consistently statistically significant, as presented in Table 2.

The 5-year follow-up showed no significant between-group differences in cardiovascular events, heart failure events, embolic stroke, or new-onset atrial fibrillation [28].

Overall, REDUCE LAP-HF I established proof of mechanism and longer-term patency, but not clinical efficacy.

#### 4.3.2. REDUCE LAP-HF II

REDUCE LAP-HF II was a randomized, double-blind, sham-controlled trial assessing the clinical efficacy of Corvia IASD in 626 patients with HFpEF or HFmrEF [9]. The primary efficacy endpoint was the hierarchical composite of cardiovascular death, non-fatal ischemic stroke at 1 year, heart failure events, and change in Kansas City Cardiomyopathy Questionnaire (KCCQ) score. A prespecified safety endpoint composite and secondary device efficacy outcomes were also evaluated.

The primary composite did not differ between groups and thus was not met in the modified intention-to-treat population. The per-protocol sensitivity analysis yielded similar neutral results, thus supporting the consistent neutrality of the trial results.

Prespecified subgroup analyses found nominal differences in treatment effects across exploratory subgroups. Male patients, patients with pulmonary artery systolic pressure at 20 W of exercise in the highest tertile (>70 mmHg) and those with right atrial volume index in the highest tertile (>29.7 mL/m^2^) demonstrated worse heart failure event outcomes with the device. A post hoc analysis identified a possible differential treatment effect according to peak exercise pulmonary vascular resistance (PVR), with a proposed threshold of 1.74 Wood units (WU) [6]. Potential benefit was observed below this value of PVR and in patients with preserved right ventricular function, while signals of worse outcomes were observed in those with elevated pulmonary pressures and right-sided remodeling.

Echocardiographic substudies across the REDUCE LAP-HF trials demonstrated predictable right atrial and right ventricular remodeling following implantation without evidence of overt right ventricular systolic dysfunction or worsening tricuspid regurgitation [8,9].

The 1-year safety composite endpoint did not differ between groups, with no significant excess in major adverse events in the device group compared with the control group. Regarding functional status, the IASD group demonstrated a greater improvement in NYHA class than the sham-control group. However, changes in KCCQ-OSS did not differ significantly between groups, as demonstrated in Table 2.

At the 3-year follow-up, the primary outcome remained neutral [28].

REDUCE LAP-HF II therefore failed to demonstrate clinical efficacy in an unselected population and shifted the field toward investigation of prospective patient phenotype selection.

#### 4.3.3. RELIEVE-HF

RELIEVE-HF was a randomized, double-blind, sham-controlled trial that evaluated the V-Wave Ventura shunt device (second generation). Participants of the trial were either HFmrEF/HFpEF or HFrEF patients [10].

The trial incorporated a primary safety outcome regarding device-related or periprocedural major adverse events at 30 days. The primary effectiveness endpoint was a hierarchical composite including all-cause mortality, various cardiac interventions, heart failure progression and change in KCCQ scores through 2 years post-implantation.

A total of 508 patients were randomized: 250 in the treatment and 258 in the control arm. Regarding the primary safety outcome, no significant major adverse events were observed. The trial demonstrated no significant difference in the primary effectiveness endpoint, indicating overall neutral results in the study population. NYHA functional class and quality-of-life parameters were also evaluated as secondary outcomes (Table 2) but should be interpreted in the context of the neutral overall primary endpoint.

Prespecified subgroup analyses indicated a varying clinical response to shunt implantation depending on left ventricular ejection fraction. Adverse cardiovascular events were observed at a greater annualized rate in the HFmrEF/HFpEF subgroup when compared with the control arm. In contrast, there were fewer adverse cardiovascular events in the HFrEF group compared with control.

**Table 2:** Exercise capacity (6MWD), NYHA functional class and quality-of-life parameters across IASD randomized trials. Values are reported as median (IQR) or mean ± SD, as specified. Unless otherwise indicated, comparisons are IASD versus sham/control. Negative changes in NYHA class indicate improvement. KCCQ-OSS, Kansas City Cardiomyopathy Questionnaire—Overall Summary Score; EQ-5D, EuroQol 5-Dimension questionnaire; 6MWD, 6 min walk distance; IQR, interquartile range; CI, confidence interval; OR, odds ratio.

Trial and Population	Follow-Up	Exercise Capacity	NYHA Class	Quality-of-Life Parameters
REDUCE LAP-HF I (n = 44; LVEF ≥ 40%)	12 months	12 months: median change in 6MWD +16 m [IQR: −57, 30] versus +13.6 m [IQR: −10, 72]; *p* = 0.31	Median improvement of 1 class in IASD arm [IQR: −1, 0] vs. 0 in control arm [IQR: −1, 0]; *p* = 0.08	KCCQ-OSS: +10.5 [IQR: 0.7, 18.8] versus +8.1 [IQR: −5.7, 20.6]; *p* = 0.57 for baseline-adjusted 12-month comparison EQ-5D: 0.0 [IQR: −0.2, 0.1] versus 0.0 [IQR: −0.1, 0.2]; *p* = 0.81 for baseline-adjusted 12-month comparison
REDUCE LAP-HF II (n = 626; LVEF ≥ 40%)	12 months	Not reported	Median improvement of 0.5 class in IASD [IQR: −1, 0] vs. 0 [IQR: −1, 0]; *p* = 0.006	KCCQ-OSS: +10.2 [IQR: −1.8, 26.8] versus +9.4 [IQR: −2.1, 22.9]; *p* = 0.73
REDUCE LAP-HF II (same as above)	24 months	Not reported	Greater likelihood of improvement by ≥1 class with IASD (OR 1.66, 95% CI 1.15–2.42; *p* = 0.01)	KCCQ-OSS: +12.1 (95% CI 9.4–14.8) versus +9.1 (95% CI 6.6–11.6); *p* = 0.09
RELIEVE-HF (n = 508; any LVEF)	12 months	Not reported	Not reported	KCCQ-OSS: mean change +9.4 ± 21.6 versus +10.2 ± 21.3; adjusted difference −0.6 points (95% CI −4.4 to 3.1); *p* = 0.74

A subsequent mechanistic analysis found left ventricular reverse remodeling without significant right-sided enlargement in HFrEF, whereas HFpEF was characterized by enlargement of the right atrium, right ventricle, and inferior vena cava and an increase in pulmonary artery systolic pressure [29]. These findings provide biological context but do not convert a neutral overall trial into definitive subgroup efficacy.

### 4.4. Synthesis of Evidence

Favorable hemodynamic effects of IASD implantation, particularly reductions in PCWP, have been consistently demonstrated across meta-analyses, including early pooled analyses [30,31]. Improvements in exercise capacity and quality of life have also been reported, but with considerably greater variability across studies. Changes in right atrial pressure, pulmonary artery pressure, and right ventricular remodeling are inconsistent across studies, suggesting a possible, but not definitively established, redistribution of preload to the right heart.

Subsequent meta-analyses incorporating randomized trial data have aligned with the neutral findings of these trials, failing to demonstrate significant reductions in hard clinical outcomes, like heart failure hospitalizations and mortality [32]. Notably, one pooled analysis suggested a potential signal toward increased heart failure events and cardiovascular mortality in HFpEF populations post IASD implantation [33], which is congruent with the RELIEVE-HF trial’s findings.

Overall, substantial heterogeneity is present across meta-analyses, reflecting differences in study designs, patient populations and device types. These inconsistencies highlight the need for careful heart failure phenotyping and patient selection in the evaluation of interatrial shunting.

#### The Promising Exploratory “Responder” Phenotype

**Figure 3:**
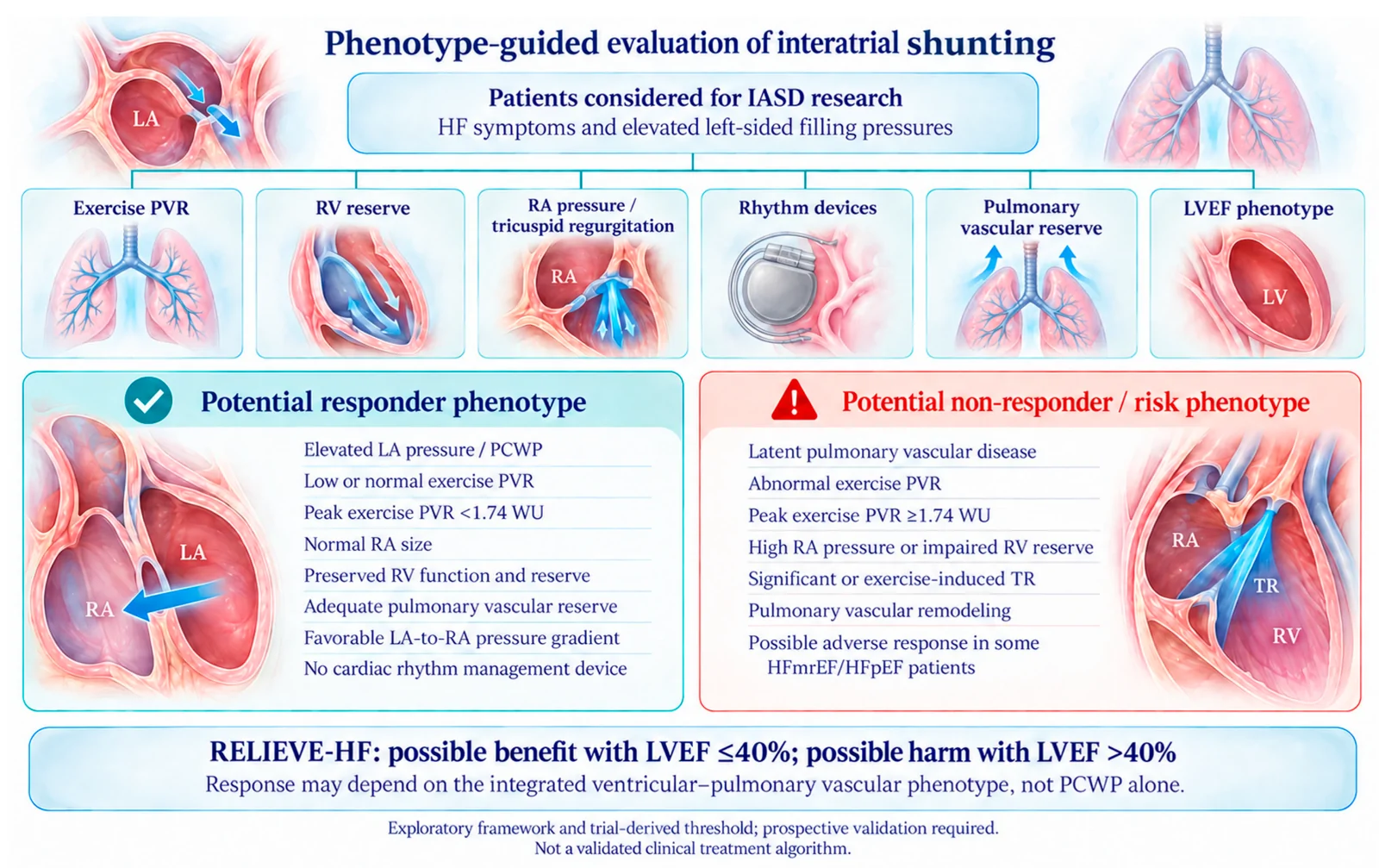
Proposed phenotype-based framework for response to interatrial shunt therapy. A potential responder profile combines elevated left-sided filling pressure with low pulmonary vascular resistance (PVR), preserved right ventricular (RV) reserve, and adequate pulmonary vascular accommodation. In contrast, patients with latent pulmonary vascular disease, elevated exercise pulmonary vascular resistance, impaired RV reserve or RV–pulmonary arterial coupling, high right atrial pressure, significant tricuspid regurgitation (TR), and cardiac rhythm management devices may be less suitable candidates for interatrial shunting. Thresholds and subgroup effects are exploratory and must not be used as validated treatment criteria.

Post hoc analyses [34] and subsequent reports [28] identified a possible “responder” subgroup among REDUCE LAP-HF II participants, which may derive greater benefit from IASD implantation in terms of heart failure events and quality of life. Patients with peak exercise pulmonary vascular resistance (PVR) <1.74 WU and no cardiac rhythm management device or implantable cardioverter defibrillator demonstrated more favorable outcomes. More specifically, fewer heart failure events and greater improvement in quality-of-life scores were observed among “responders”, as well as a higher incidence of ischemic stroke and a lower incidence of worsening kidney function. The proposed phenotype-based framework derived from these exploratory analyses and subsequent responder investigations is illustrated in Figure 3. However, the peak exercise PVR threshold of 1.74 WU derived from post hoc analyses of REDUCE LAP-HF II, as well as the absence of cardiac rhythm device remain exploratory criteria. This should not be presented as a validated diagnostic boundary or verified implantation criterion. It is an unverified hypothesis currently undergoing prospective evaluation in the RESPONDER-HF trial (NCT05425459). Enrollment ended with 266 patients in May 2026 and clinical validation remains pending.

## 5. Discussion

The cumulative evidence on IASDs demonstrates a consistent and reproducible positive physiological effect, primarily expressed as reductions in left atrial pressure and PCWP at rest and, more prominently, during exercise. This hemodynamic signal has been consistently observed in early feasibility and non-randomized studies, as well as in pooled analyses that supported acceptable periprocedural safety. In contrast, symptomatology, exercise capacity and quality-of-life measurements are less consistent across studies. The overall encouraging early hemodynamic response should be interpreted cautiously, given the studies’ small sample sizes, predominantly single-arm and non-randomized designs, limited blinding, and susceptibility to placebo effects.

Despite this physiological signal, subsequent RCTs failed to demonstrate consistent clinical benefit in unselected HFpEF/HFmrEF populations, as reflected in hard outcomes, like heart failure hospitalizations and mortality. This discrepancy between promising early feasibility data and larger, sham-controlled randomized trial outcomes represents one of the central issues in the field. In REDUCE LAP-HF II, interatrial shunting did not improve the primary hierarchical composite endpoint in the overall population, despite prior evidence of favorable hemodynamic effects. Similarly, RELIEVE-HF showed overall neutral results, albeit with important heterogeneity across heart failure phenotypes. These findings suggest that reduction in left-sided filling pressure alone may be insufficient to improve clinical outcomes across the broad and physiologically heterogeneous spectrum of heart failure phenotypes.

One possible explanation for this divergence is that the clinical effect of IASD therapy may be strongly influenced by baseline hemodynamic phenotype, pulmonary vascular reserve, and right ventricular adaptation. The responder phenotype proposed following REDUCE LAP-HF II is therefore particularly important, although it remains exploratory and should not yet be interpreted as definitive evidence of efficacy. Post hoc analyses suggested that patients with lower peak exercise PVR, commonly defined as PVR <1.74 Wood units, and with no cardiac rhythm management device may derive greater benefit from shunt implantation, as Figure 3 suggests. This subgroup may represent patients among whom elevated left-sided filling pressure can be effectively decompressed without imposing excessive afterload or volume burden on the right heart.

Conversely, patients with the distinct hemodynamic phenotype of HFpEF with latent pulmonary vascular disease (latent PVD) [35] may be less suitable candidates for IASD therapy. This phenotype corresponds to a normal or near-normal PVR at rest, which becomes abnormally elevated during exercise (PVR ≥1.74 WU at peak exercise). Latent PVD is characterized by preserved resting PVR with an abnormal increase during exercise, reflecting impaired pulmonary vascular reserve before resting PVD becomes apparent [6]. In HFpEF, retrograde transmission of high left ventricular filling pressure to the pulmonary circulation leads to post-capillary pulmonary hypertension, which in turn may be the cause of pulmonary vascular remodeling and potential development of increased PVR in predisposed HFpEF patients [36]. Furthermore, the prevalence of latent PVD in HFpEF populations has been estimated at approximately 20–30% and is associated with greater disease severity [37]. This phenotype has also been found to correlate with tricuspid regurgitation, which is commonly uncovered during exercise, mirrored by congruent steep increases in right atrial pressure. On this basis, IASD therapy may be less favorable in patients with latent PVD HFpEF, which should be carefully considered and prospectively evaluated in future trials. Accordingly, possible clinical benefit may be more consistently demonstrated among patients with satisfactory pulmonary vascular reserve and right ventricular function. It should be considered that latent PVD can only be evaluated by invasive hemodynamic testing during exercise, which has not been uniformly applied across IASD trials. A prespecified substudy within the ongoing RESPONDER-HF trial [38] aims to determine whether exercise echocardiographic parameters can serve as noninvasive predictors of PVD and treatment response.

The right ventricular functional reserve, as previously mentioned, represents another potentially important determinant of IASD tolerability. Assessment of right ventricular capacity should extend beyond resting systolic function and incorporate RV–pulmonary artery coupling, diastolic compliance, and hemodynamic adaptation during exercise [29]. Echocardiographic parameters, including TAPSE, RV fractional area change, RV free-wall strain, and the TAPSE/PASP ratio, may provide complementary information on RV systolic function and RV–pulmonary artery coupling. Invasive exercise hemodynamic assessment via right-heart catheterization may help characterize changes in RA pressure, pulmonary artery pressure, cardiac output, and PVR during exercise. Hemodynamic reserve during exercise is a potential determinant of the right heart’s ability to accommodate the shunt flow, especially under physiological stress. However, it should be noted that no individual echocardiographic or invasive parameter has yet been validated as a definitive predictor of IASD-related right-heart intolerance.

In the RELIEVE-HF trial, the evaluation of left ventricular ejection fraction (LVEF) in correlation to total cardiovascular events demonstrated improved outcomes post IASD implantation among patients with an LVEF ≤ 40% (HFrEF) and a simultaneously worsened prognosis among HFmrEF/HFpEF patients with LVEF > 40%. These findings [10] challenge the initial premise that interatrial shunting would be most effective in HFpEF patients. Conversely, the trial suggested that the physiological consequences of left-to-right shunting may contribute to an increase in pulmonary artery systolic pressure, right ventricular end-diastolic volume, and inferior vena cava diameter. These findings suggest increased right ventricular preload and pulmonary vascular loading, potentially resulting in adverse right-heart remodeling in patients with limited right ventricular reserve. In contrast, patients with HFrEF may have greater capacity to accommodate the redistribution of preload following shunt implantation. SUSTAIN-HF provides concordant but uncontrolled observations in an HFrEF population [26]. It should be mentioned that RELIEVE-HF enrolled a population with a greater burden of comorbidities than REDUCE LAP-HF II, which may have contributed to the observed heterogeneity of outcomes.

Based on the RELIEVE-HF mechanistic analysis, LV and RV diastolic compliance were found to be lower at baseline in HFpEF compared with HFrEF patients. Following shunt implantation, HFpEF patients showed further adverse changes in RV, whereas comparable changes were not observed in the HFrEF group. Thus, differences in ventricular stiffness and chamber compliance may contribute to the heterogeneous response to interatrial shunting. In HFpEF, increased LV stiffness and impaired diastolic reserve contribute to disproportionate elevations in exercise filling pressures. Concomitant impairment of LA compliance may further amplify the pressure response to increased pulmonary venous return. Although LA decompression may reduce pulmonary venous pressure, the extent to which this translates into improved ventricular filling and exercise capacity may depend on the underlying ventricular and atrial pressure–volume relationships. The independent contribution of LA compliance, as distinct from LV stiffness and RV compliance, remains insufficiently characterized. Further mechanistic studies incorporating direct or validated estimates of chamber compliance, atrial mechanics, and exercise hemodynamics may help clarify the interaction between left-sided unloading and right-heart tolerance.

Moreover, the potential influence of ventricular phenotype on the response to interatrial shunting warrants further consideration. Although HFpEF and HFmrEF are frequently grouped together in IASD trials, these phenotypes may differ in ventricular remodeling, systolic reserve, diastolic properties, and right-heart adaptation. Importantly, the available randomized evidence does not yet establish whether patients with HFmrEF and HFpEF exhibit distinct responses to interatrial shunting, as most trials have either combined these populations or compared patients with LVEFs below 40% to those above this threshold. Future studies incorporating prespecified LVEF-stratified analyses are required to clarify whether the LVEF phenotype is an independent determinant of treatment response.

Despite sharing the common objective of reducing LA pressure, left atrial decompression technologies should not be treated as hemodynamically interchangeable. IASDs differ in architecture, fenestration size, and shunt configuration, which may influence flow dynamics and RV loading. The Corvia IASD uses a fixed 8 mm fenestration, whereas the Occlutech AFR offers variable fenestration sizes, allowing for greater flexibility in shunt sizing. A larger orifice may permit greater flow under comparable pressure-gradient and resistance conditions, but the actual shunt flow and Qp/Qs are also influenced by the interatrial pressure gradient and the patient’s cardiopulmonary hemodynamics. The V-Wave system has evolved from a valved design, intended to restrict reverse flow, to a valveless configuration following concerns regarding valve-associated tissue overgrowth and shunt narrowing. In contrast, the APTURE system creates LA-to-coronary sinus communication, providing an alternative pathway for left atrial decompression and right-sided flow. These structural differences may result in distinct patterns of left atrial unloading and right ventricular volume loading. Overall, the device architecture, fenestration size, and shunt location represent plausible determinants of the balance between LA unloading and right-sided volume loading; however, their comparative hemodynamic and clinical implications remain insufficiently established. This emphasizes the need for device-specific evaluation and direct comparative studies rather than assuming a uniform class effect.

**Figure 4:**
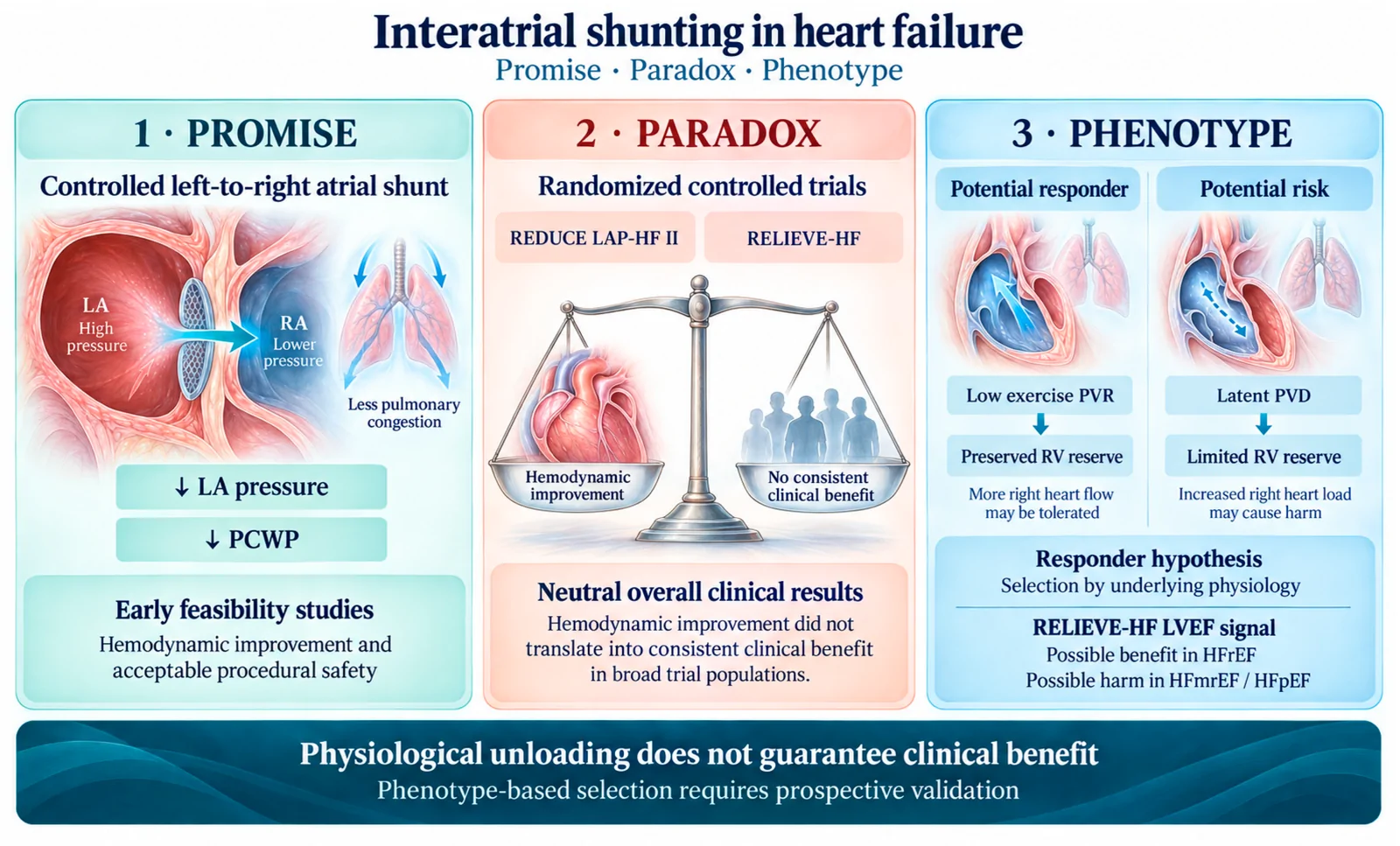
Evolution of the current understanding of interatrial shunt therapy. Early studies consistently demonstrated favorable hemodynamic effects (“Promise”), whereas subsequent randomized controlled trials showed neutral overall clinical outcomes despite successful left atrial decompression (“Paradox”). Contemporary evidence increasingly supports a phenotype-guided approach to patient selection, emphasizing pulmonary vascular reserve, right ventricular adaptation, and exercise hemodynamics (“Phenotype”).

The available evidence suggests that while IASDs achieve their intended physiological effect, their clinical utility is limited and potentially deleterious in unselected populations. Collectively, the findings underscore the role of heart failure phenotype and hemodynamic profile as determinants of both efficacy and safety, while also highlighting the need for phenotype-specific evaluation and targeted application of IASDs. Future trials should move beyond the simple identification of elevated PCWP and incorporate comprehensive hemodynamic phenotyping, including assessment of pulmonary vascular reserve, right ventricular function, exercise hemodynamics, rhythm-device status, and potentially LVEF-defined response patterns. Because invasive exercise hemodynamics remain the reference standard for identifying latent PVD, an important unresolved question is whether noninvasive imaging or exercise echocardiographic markers can reliably identify the same physiological phenotype and guide patient selection. Until phenotype-specific benefit is prospectively demonstrated, IASD therapy should remain investigational rather than routinely applied to unselected HFpEF/HFmrEF populations. The conceptual evolution of interatrial shunt therapy, from physiological promise to phenotype-guided investigation, is summarized in Figure 4.

## 6. Conclusions and Future Directions

IASDs represent one of the most physiologically compelling device-based approaches developed for the treatment of HFpEF/HFmrEF. They consistently reduce left atrial pressure and PCWP and have demonstrated procedural feasibility and acceptable short-term safety in experienced centers. Nevertheless, randomized evidence has not shown consistent overall clinical benefit in unselected HFpEF/HFmrEF populations, and emerging data suggest that some phenotypes may derive no benefit or potentially experience harm.

In the broader spectrum of rapidly evolving heart-failure therapeutics, including pharmacological and device-based strategies, the clinical value of interatrial shunting remains uncertain. The next phase of investigation should prioritize phenotype-driven trial design, rigorous invasive or validated noninvasive hemodynamic characterization, and device-specific evaluation rather than assuming class equivalence across shunt platforms.

At present, IASDs should be regarded as investigational therapies whose role in routine clinical practice remains undefined. Further randomized studies focused on carefully selected patient populations are required before interatrial shunting can be considered an established therapeutic strategy in heart failure management. IASDs may ultimately prove not to be a failed therapy, but rather a therapy awaiting identification of the appropriate patient phenotype.

## Figures and Tables

**Table 1:** Interatrial shunt technologies evaluated or under investigation in heart failure.

Device (Manufacturer)	Design Characteristics	Fenestration Diameter	Primary Intended Population	Highest Level of Clinical Evidence	Key Studies	Take-Home Message
Corvia IASD (Corvia Medical)	Fixed nitinol shunt	8 mm	HFpEF/HFmrEF	Large sham- controlled RCT	REDUCE LAP-HF REDUCE LAP-HF I REDUCE LAP-HF II	Exercise PCWP ↓ Neutral pivotal RCT Responder phenotype under investigation
Occlutech AFR (Occlutech)	Fenestrated double-disk occluder	6–10 mm (commonly 8 mm)	HFpEF/HFrEF, PAH, congenital heart disease, restrictive cardiomyopathy	Prospective studies + registries	AFR-PRELIEVE FLOW-HF Registry AFTER Registry	Symptom improvement + Exercise capacity ↑ Based on observational evidence only
V-Wave (V-Wave Ltd.)	Valved—1st generation Valveless—2nd generation	~5 mm	Initially HFrEF, later broader HF	Early multicenter + RCT	Early feasibility trials RELIEVE-HF program	1st-generation: high valve stenosis rates 2nd-generation redesign Benefit mainly in HFrEF under investigation
Alleviant (Alleviant Medical)	Implant-free septostomy	~7 mm	HFpEF (implant- free concept)	Early feasibility	Early feasibility trials	Implant-free concept Early feasibility
APTURE (Edwards Lifesciences)	Permanent implant	~7 mm	HFpEF	Early feasibility	Early feasibility studies	Limited clinical experience

AFR, Atrial Flow Regulator; IASD, interatrial shunt device; HFpEF, heart failure with preserved ejection fraction; HFmrEF, heart failure with mildly reduced ejection fraction; RCT, randomized controlled trial; PCWP, pulmonary capillary wedge pressure; PAH, pulmonary arterial hypertension; HFrEF, heart failure with reduced ejection fraction; HF, heart failure. ↑, increase; ↓, decrease.

## Data Availability

No new data was created or analyzed in this study. Data sharing is not applicable to this article.

## Supplementary Materials

The following supporting information can be downloaded at: https://www.mdpi.com/article/10.3390/jcdd13100519/s1, Supplementary Figure S1. Structural designs and configurations of interatrial shunt devices investigated for the treatment of heart failure. The panels are conceptual, not to scale, and are not product photographs or engineering drawings. The figure illustrates the principal device architectures, including the Corvia interatrial shunt device (IASD), Occlutech atrial flow regulator (AFR), V-Wave shunt system, and APTURE system. Differences in fenestration size, flow-control mechanisms (valved or valveless), and shunt location are highlighted to illustrate the design characteristics that may influence shunt flow and the hemodynamic response. Schematic representations are intended to illustrate device configuration and are not to scale.

## Abbreviations

The following abbreviations are used in this manuscript:

6MWD	6-Minute Walk Distance
AFR	Atrial Flow Regulator
BNP	B-type Natriuretic Peptide
EQ-5D	European Quality of Life 5-Dimension
HF	Heart Failure
HFmrEF	Heart Failure with Mildly Reduced Ejection Fraction
HFpEF	Heart Failure with Preserved Ejection Fraction
HFrEF	Heart Failure with Reduced Ejection Fraction
IASD	Interatrial Shunt Device
KCCQ	Kansas City Cardiomyopathy Questionnaire
KCCQ-OSS	Kansas City Cardiomyopathy Questionnaire—Overall Summary Score
LA	Left Atrium/Left Atrial
LV	Left Ventricle/Left Ventricular
LVEF	Left Ventricular Ejection Fraction
NT-proBNP	N-terminal proBNP
NYHA	New York Heart Association
PAH	Pulmonary Arterial Hypertension
PASP	Pulmonary Artery Systolic Pressure
PCWP	Pulmonary Capillary Wedge Pressure
PVD	Pulmonary Vascular Disease
PVR	Pulmonary Vascular Resistance
Qp/Qs	Pulmonary-to-Systemic Blood Flow Ratio
RA	Right Atrium/Right Atrial
RCT	Randomized Controlled Trial
RV	Right Ventricle/Right Ventricular
TAPSE	Tricuspid Annular Plane Systolic Excursion
TR	Tricuspid Regurgitation
WU	Wood Units
