# Supplementary material for: Interatrial Shunt Devices in Heart Failure: A Therapy in Search of the Right Patient
Source: J Cardiovasc Dev Dis. 2026 Oct 16;13(10):519. doi: 10.3390/jcdd13100519 (PMC13705546; doi:10.3390/jcdd13100519)
Supplement: Supplementary file 1 [file jcdd-13-00519-s001.zip › jcdd-4543423-supplementary.pdf]

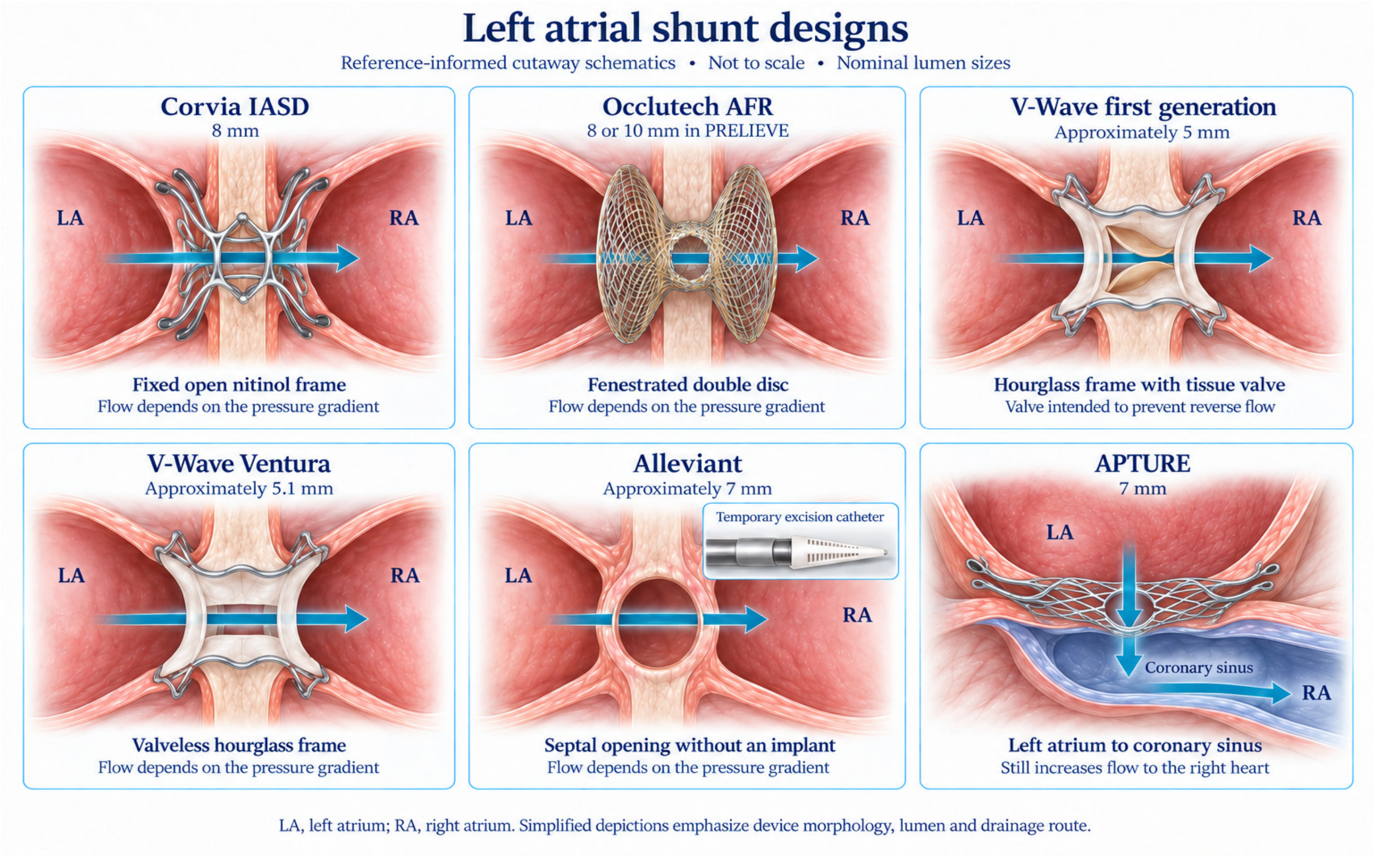

Supplementary Figure S1. Structural designs and configurations of interatrial shunt devices investigated for the treatment of heart failure. The panels are conceptual, not to scale, and are not product photographs or engineering drawings. The figure illustrates the principal device architectures, including the Corvia interatrial shunt device (IASD), Occlutech atrial flow regulator (AFR), V-Wave shunt system, and APTURE system. Differences in fenestration size, flow-control mechanisms (valved or valveless), and shunt location are highlighted to illustrate the design characteristics that may influence shunt flow and the hemodynamic response. Schematic representations are intended to illustrate device configuration and are not to scale.
